# Selenite Removal from Aqueous Solution Using Silica–Iron Oxide Nanocomposite Adsorbents

**DOI:** 10.3390/gels9060497

**Published:** 2023-06-19

**Authors:** Georgiana Mladin, Mihaela Ciopec, Adina Negrea, Narcis Duteanu, Petru Negrea, Paula Svera (m. Ianăşi), Cătălin Ianăşi

**Affiliations:** 1 Faculty of Industrial Chemistry, Environmental Engineering, Polytechnic University of Timişoara, Victoriei Square no. 2, 300006 Timişoara, Romania; 2 National Institute for Research and Development in Electrochemistry and Condensed Matter, 144th Dr. A. P. Podeanu Street, 300569 Timisoara, Romania; 3 ”Coriolan Drăgulescu” Institute of Chemistry, Bv. Mihai Viteazul, No. 24, 300223 Timisoara, Romania

**Keywords:** selenite removal, adsorption, silica matrix, nanocomposite, iron oxide, sol–gel

## Abstract

In recent years, during industrial development, the expanding discharge of harmful metallic ions from different industrial wastes (such as arsenic, barium, cadmium, chromium, copper, lead, mercury, nickel, selenium, silver, or zinc) into different water bodies has caused serious concern, with one of the problematic elements being represented by selenium (Se) ions. Selenium represents an essential microelement for human life and plays a vital role in human metabolism. In the human body, this element acts as a powerful antioxidant, being able to reduce the risk of the development of some cancers. Selenium is distributed in the environment in the form of selenate (SeO_4_^2–^) and selenite (SeO_3_^2–^), which are the result of natural/anthropogenic activities. Experimental data proved that both forms present some toxicity. In this context, in the last decade, only several studies regarding selenium’s removal from aqueous solutions have been conducted. Therefore, in the present study, we aim to use the sol–gel synthesis method to prepare a nanocomposite adsorbent material starting from sodium fluoride, silica, and iron oxide matrices (SiO_2_/Fe(acac)_3_/NaF), and to further test it for selenite adsorption. After preparation, the adsorbent material was characterized by scanning electron microscopy (SEM) and energy-dispersive X-ray spectroscopy (EDX). The mechanism associated with the selenium adsorption process has been established based on kinetic, thermodynamic, and equilibrium studies. Pseudo second order is the kinetic model that best describes the obtained experimental data. Also, from the intraparticle diffusion study, it was observed that with increasing temperature the value of the diffusion constant, K_diff_, also increases. Sips isotherm was found to best describe the experimental data obtained, the maximum adsorption capacity being ~6.00 mg Se(IV) per g of adsorbent material. From a thermodynamic point of view, parameters such as ΔG^0^, ΔH^0^, and ΔS^0^ were evaluated, proving that the process studied is a physical one.

## 1. Introduction

Selenium is a non-metal, being considered a rare element because it can be found in the Earth’s crust only in small concentrations (1.3 × 10^−5^%) [1]. Selenium is an element that has some toxicity, but at the same time represents an important micronutrient for humans and animals [2]. Selenium is used in different industries for the production of photocopies, glass, or in the production of different ceramic materials. In addition, due to all of its industrial applications, selenium can contaminate soil, air, land, municipal landfills, waste treatment plants, and agricultural land [3].

Selenium species (selenite or selenate) are toxic, but from these selenite (Se(IV)) presents higher toxicity. The dominant species in water depends on the physico-chemical factors and the redox conditions, but also on the pH, with the dominant species being ${SeO}_{3}^{2-}$ [4,5,6]. The European Commission has established the Drinking Water Regulation Limit (DWRL) for selenium as 10 µg L^−1^ [7].

There are a multitude of methods for removing Se(IV) from aqueous solutions, such as filtration [8], nanofiltration [9], reverse osmosis [10], co-precipitation [11], ion exchange [12], oxidation/reduction [8], electrocoagulation [13], electrochemical reduction [14], photocatalysis using TiO_2_ [15], advanced reduction with perchlorate [16,17] or with 1,2-dichloroethane [18,19], precipitation [20], biological treatment using microbial reduction [12] and bioreactors [21], phytoremediation [22], and also adsorption [8]. Some removal techniques generate large amounts of sludge, others are quite expensive, and some others do not have the desired efficiency. However, from all these techniques, a special importance is given to adsorption. It is known that in the adsorption process, an important role is played by the materials used as adsorbents. Based on the literature data, it was found that selenium was recovered by using different adsorbent materials, such as magnetic nanoparticles–graphene oxide (MGO) [8,23], modified biochar [8,24], ZnO-based nanocomposites [8,25], activated carbon coated with copper (Cu-AC) [8,26,27], oxi-iron hydroxides (FeOOHs) [28], carbon nanotubes with zero valence iron [29], and activated alumina [30].

The main objective of current investigation is to eliminate Se(IV) ions from aqueous solution through adsorption using a nanocomposite adsorbent material. The desired adsorbent material was prepared via sol–gel synthesis using a combination of silica matrix, iron oxide, and NaF (SiO_2_/Fe(acac)_3_/NaF) as precursors. Prepared Fe (III)-based nanoparticles present a large number of beneficial properties, such as affordability, good environmental stability even under extreme conditions, and a polar surface structure. All these properties make them suitable for a large number of applications, such as catalysis and as materials used for the development of data storage devices and environmental remediation systems [31,32,33].

From all these possible applications, Se(IV)’s removal by adsorption was achieved due to the adsorbent material’s surface area and porosity, which can be manipulated by the synthesis parameters. To the best of our knowledge, newly prepared adsorbent material have not yet been studied for Se(IV) recovery, emphasizing the novelty of the present research and its potential efficacy for adsorptive processes.

## 2. Results and Discussion

### 2.1. Scanning Electron Microscopy, SEM

**Figure 1:**
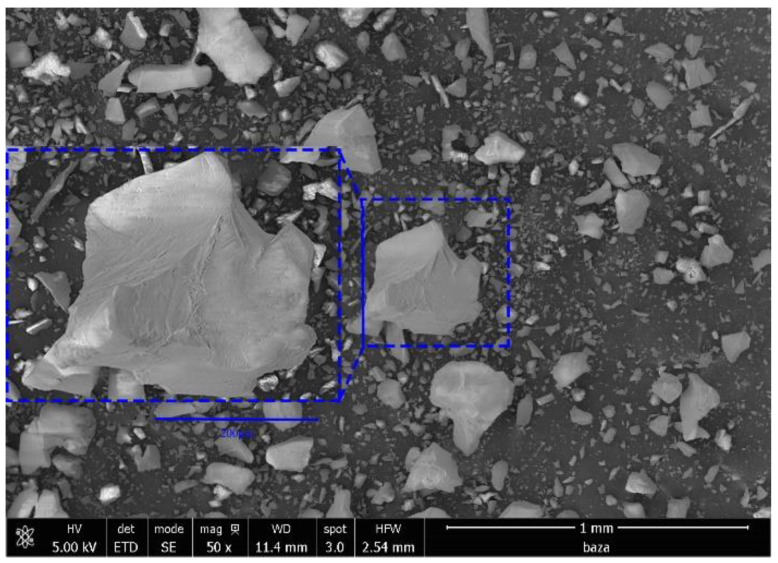
Scanning electron microscopy image obtained for prepared SiO_2_/Fe(acac)_3_/NaF material.

In order to investigate the surface morphology of the synthesized material, scanning electron microscopy, SEM, at different magnifications (500×, 250×, 100×, and 50×) was performed, and the obtained images are presented in Figure 1.

**Figure 2:**
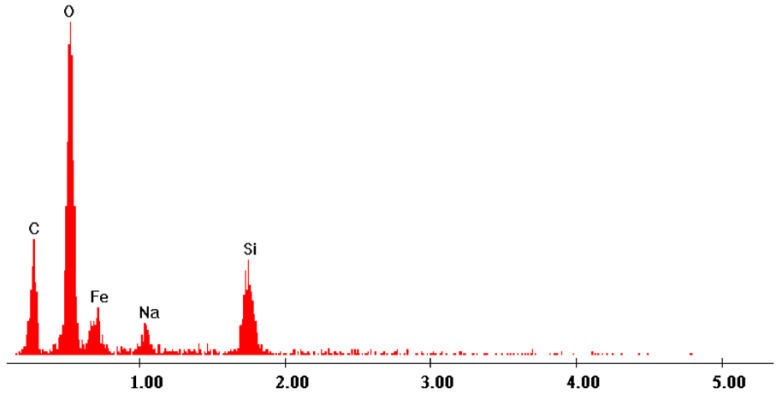
Energy-dispersive X-ray spectrum recorded for prepared SiO_2_/Fe(acac)_3_/NaF material.

By analyzing the images depicted in Figure 1, one can observe that the synthesized adsorbent material presenst a higher degree of crystallinity. From all these images, one can observe the presence of crystals with different shapes and sizes (mean size of these crystals being around 50 μm). Further, in order to determine the elemental composition of the prepared material, the energy-dispersive X-ray spectrum (EDX) was recorded, which is presented in Figure 2. Based on the recorded spectrum, the elemental composition of the material was determined (Table 1).

From the data depicted in Table 1, one can observe the presence of Fe, O, and Si atoms, which confirms the formation of the SiO_2_/Fe(acac)_3_ matrix. The presence of Na and F is also attributed to the use of NaF as a loosener agent during synthesis. The presence of C may also be associated with the incomplete synthesis of acetylacetonate. 

### 2.2. Selenium Adsorption Studies

#### 2.2.1. Kinetic and Thermodynamic Studies

**Figure 3:**
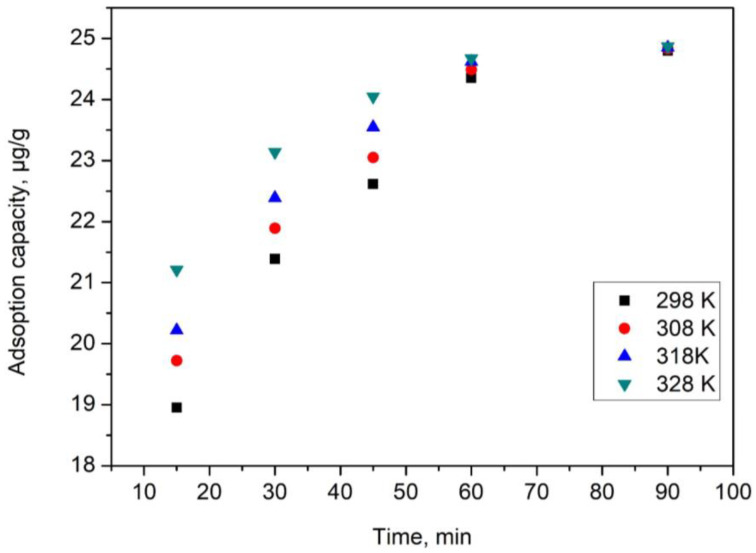
Effect of contact time and temperature on the adsorption capacity.

It is well known that all adsorption processes are influenced by the contact time and temperature. Such parameters can represent some limiting factors for all practical applications of the adsorption process. In Figure 3, the experimental data are depicted, which show the influence of contact time and temperature in the studied adsorption case.

From the information displayed in Figure 3, it can be seen that as the contact time increases, the adsorption capacity has a similar behavior. This behavior is observed until the contact time reaches 60 min, when the adsorption capacity remains approximately constant, so any further contact time increase is not justified. Based on the observation that the temperature increase leads to an increase in the adsorption capacity, one can conclude that the Se(IV) adsorption is influenced by temperature; however, observing that the adsorption capacity does not have significant increase, any further studies are not justified to be performed at temperatures over 298 K.

#### 2.2.2. Kinetic Studies

**Figure 4:**
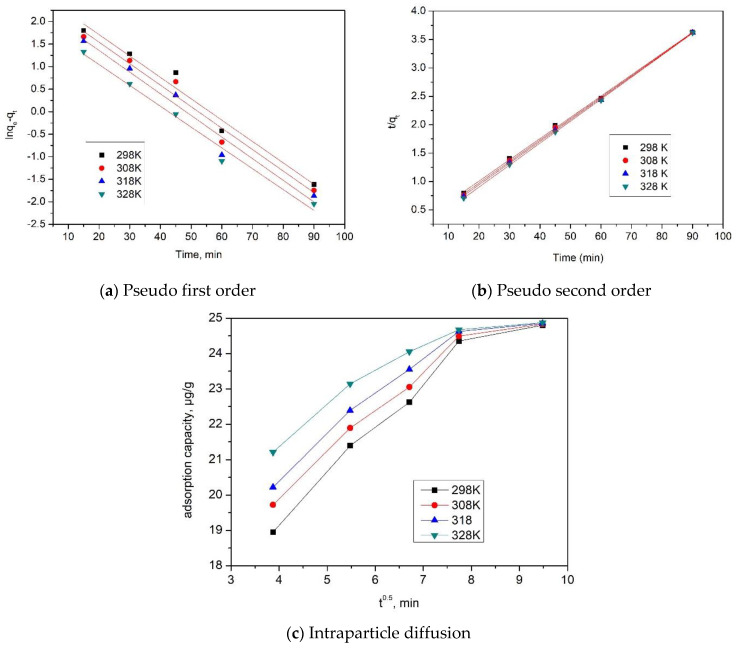
Performed kinetic studies for Se(IV) adsorption on prepared adsorbent material.

Further, in order to explain adsorptive process kinetics, experimental data were modeled by using pseudo-first-order and pseudo-second-order models, and in order to determine if film diffusion or intraparticle diffusion is the determinant stage of adsorption speed, experimental data were modeled using the Weber and Morris model. The obtained information is displayed in Figure 4.

By taking into account the kinetic parameters estimated for each kinetic model, it was possible to determine which model better described the adsorption of Se(IV) ions onto the prepared adsorbent material, based on the values of the correlation coefficient, R^2^.

The mechanism of Me^n+^ adsorption can be established by finding out if the process takes place in one or in several stages. This was established by graphically representing q_t_ = f(t^1/2^) at the three operating temperatures, and the parameters K_diff_ and C were determined.

The speed constants, calculated adsorption capacity, as well as the values obtained for the parameters K_diff_ and C by applying the kinetic models are depicted in Table 2, along with the values of the regression coefficient, R^2^.

From the information displayed in Table 2, we can conclude that the pseudo-second-order kinetic model better describes the obtained experimental data, this conclusion being supported by the almost unity value of the regression coefficient R^2^. Moreover, the adsorption capacity (q_e,calc_) value estimated from the pseudo-second-order model has a closer value to the experimental determined adsorption capacity (q_e,exp_). Also, one can observe that the temperature increase does not have a significant impact on the values of estimated parameters k_2_, q_e,calc_, so one can conclude that it is not essential to work at temperatures higher than 298 K. Simultaneously, it can be observed that Se(IV) adsorption takes place in a few stages, because the linear dependence between q_t_ and t^1/2^ does not pass through the origin for all studied temperatures. Hence, it is possible to say that intraparticle and film diffusion impact Se(IV) adsorption kinetics.

From the information displayed in Table 2, one can observe that the temperature impacts the value of the K_diff_ parameter. It is also observed that the diffusion constants specific to stage 1 are higher than the diffusion constants specific to stage 2. From this observation, we can conclude that the speed determinant stage is stage 2 [34].

#### 2.2.3. Thermodynamic Studies

The determination of the thermodynamic parameters of the adsorption process of Se(IV) on the SiO_2_/Fe(acac)_3_/NaF material was performed in the temperature range 298–328 K. From the values of these parameters, we can obtain information related to the spontaneity of the adsorption process and establish whether or not the process is influenced by temperature.

The adsorbent performance of materials is described by thermodynamic variables of the variation in enthalpy (ΔH°), entropy (ΔS°), and Gibbs free energy (ΔG°), which help to establish the adsorption mechanism.

**Figure 5:**
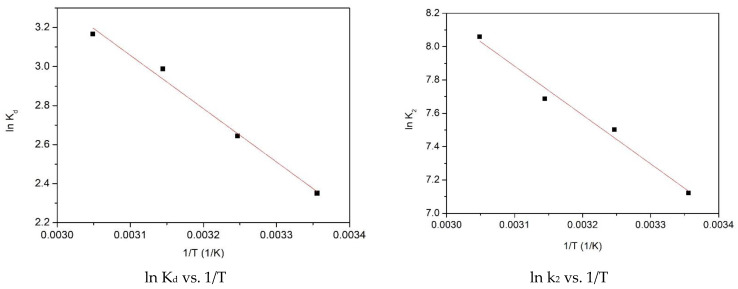
Thermodynamic studies performed for Se(IV) adsorption on prepared adsorbent material.

Representing lnK_d_ = f(1/T) graphically, a line was obtained (Figure 5), from the slope of which the values of the variations of free entropy and free enthalpy were obtained, and by means of the van’t Hoff equation the variation in Gibbs free energy was calculated, with data presented in Table 3.

Table 3 shows the thermodynamic parameters resulting from the three temperatures.

From the information displayed in Table 3, one can observe the positive value of ΔH^0^, which is associated with an endothermic adsorptive process. The negative value of ΔG^0^ increases with the temperature increase, indicating that the studied adsorptive process is a spontaneous one and is influenced by temperature. The positive value of ΔS^0^ is associated with a favorable adsorptive process at the adsorbent/adsorbate interface. The activation energy has a value of 8.3 kJ mol^−1^ and, because this value is lower than 40 kJ mol^−1^, we can conclude that the studied adsorption process has a physical nature [35].

#### 2.2.4. Equilibrium Studies

To describe how Se(IV) interacts with the SiO_2_/Fe(acac)_3_/NaF material, equilibrium studies were performed, consisting of the mathematical modeling of obtained experimental data by using the Langmuir, Freundlich, and Sips adsorption isotherms. The correlation coefficient, R^2^, was also determined in order to find the isotherm that best describes the Me^n+^ ion adsorption process.

**Figure 6:**
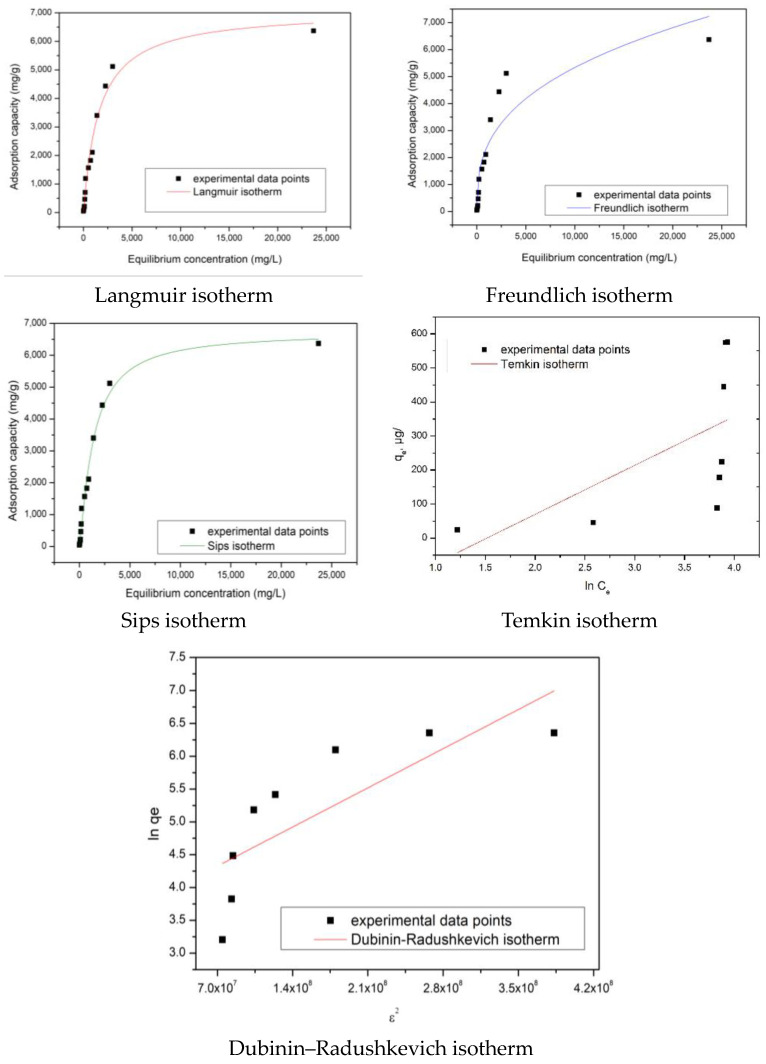
Equilibrium studies performed for Se(IV) adsorption on prepared adsorbent material.

Figure 6 depicts the obtained adsorption isotherms associated with Se(IV) adsorption on the SiO_2_/Fe(acac)_3_/NaF material.

The particular parameters associated with each isotherm used to model experimental data were evaluated from the slopes of the linear form of each isotherm and using the ordinate from the origin (Table 4).

The interdependence established between the Se(IV) equilibrium concentration and the adsorption capacity illustrate that an increase in the equilibrium concentration leads to an increase in the adsorption capacity, until an equilibrium is reached, corresponding to the experimental obtained maximum adsorption capacity (qe,exp).

Based on the information presented in Table 4, it is observed that the Se(IV) adsorption on the prepared adsorbent material better fits the Sips isotherm, due to the value of the regression coefficient being almost 1. In addition, one can observe that the calculated value of the adsorption capacity is closer to the experimentally determined one.

From the information found in the scientific literature, we are able to provide a comparison of the adsorption capacities of different adsorbent materials, and compare them with the adsorption capacity of the SiO_2_/Fe(acac)_3_/NaF. From the information presented in Table 5, we can conclude that SiO_2_/Fe(acac)_3_/NaF presents a good adsorption capacity.

#### 2.2.5. pH Effect

**Figure 7:**
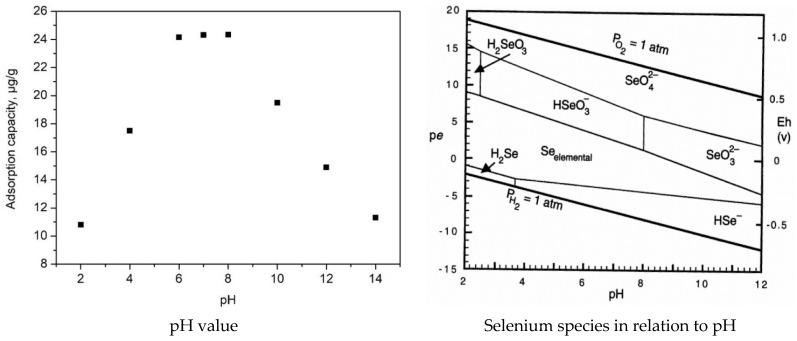
pH effect on the Se(IV) adsorption and Se(IV) species in relation to pH [48].

Figure 7 shows how the adsorption capacity of Se(IV) is influenced by the evolution of pH.

In this way, we can observe that at a pH lower than 6 the adsorption capacity increases with the increase in the pH. When the pH is in the range of 6 to 8, it is observed that the adsorption capacity reaches maximum values (~24 µg Se(IV) g^−1^ material). This behavior can be explained only by taking into account the value of pH-pZc (presented in [32]). In this pH range, the adsorbent surface is positively charged, allowing the adsorption of anionic species from the solution. The experimental data proved that stable Se species at this pH is HSeO_3_^–^, which presents a higher affinity for Fe atoms from adsorbent material. Further, when the pH is higher than 8, the adsorption capacity decreases. According to Section 4.1, the species of selenium that can be adsorbed on the surface of the material are ${HSeO}_{3}^{-}$ or ${SeO}_{3}^{2-}$ [48].

Based on this information, a possible mechanism has been proposed. In the first step, we must consider that the aqueous medium γFe_2_O_3_ undergoes a superficial hydrolysis process with the formation of iron oxo-hydroxides. Further, HSeO_3_^–^ or SeO_3_^2–^ will be linked onto the material surface by attraction forces established between these negative charges and hydrogen atoms from superficial iron oxo-hydroxides.

## 3. Conclusions

In our study, a new adsorbent material was prepared based on SiO_2_, Fe(acac)_3_, and NaF as a porogenic agent, with the preparation of the desired adsorbent material being proved based on physical-chemical analysis. After that, the obtained material was tested as an adsorbent for Se(IV) removal from aqueous solutions.

The recorded data proved that the Se(IV) adsorption is dependent on solution pH, with an optimum pH range between 7 and 8. Based on the recorded experimental data, kinetic, thermodynamic, and equilibrium studies were performed. Kinetic studies demonstrated that the Se(IV) adsorption is better described by the pseudo-second-order model. The Weber–Morris model proved that the porous structure of the SiO_2_/Fe(acac)_3_/NaF adsorbent material also favors the placement of the active adsorption sites on the interior surface of the adsorbent channels. Such active site distribution indicates that the Se(IV) ions are adsorbed on the material surface in the first step, and in the second step the adsorption process reaches an equilibrium, meaning that the intraparticle diffusion is not at the limiting stage. Based on these observations, we can conclude that Se(IV) adsorption takes place as a film adsorption. From the value of activation energy, we can conclude that the studied adsorption process is a physical one.

Thermodynamic studies confirm that Se(IV) adsorption is an endothermic and spontaneous process, being influenced by temperature. Also, it was confirmed that the adsorption process takes place at the adsorbent material/solution interface. Following the equilibrium studies, the Sips model is the one that best describes the adsorption process, establishing the maximum adsorption capacity of the SiO_2_/Fe(acac)_3_/NaF material as ~6 mg Se(IV) g−1 material. A comparative study with data from the literature regarding the affinity of the synthesized material, SiO_2_/Fe(acac)_3_/NaF, for selenium, has been shown to be significant.

## 4. Materials and Methods

### 4.1. Synthesis and Characterization of SiO_2_/Fe(acac)_3_/NaF Material

The present research paper reports the synthesis of a (SiO_2_/Fe(acac)_3_/NaF) nanocomposite via sol–gel method, as outlined in reference [32]. Sol–gel synthesis represents an effective method to obtain materials with desired surfaces. One important advantage of sol–gel synthesis is the possibility to obtain materials with higher surface areas [32].

For adsorbent material preparation, the following materials were used: Fe (III) acetylacetonate, Fe(acac)_3_ (Sigma Aldrich, St. Louis, MO, USA); methanol (Chimopar, SC CHIMOPAR TRADING SRL, București, Romania); tetraethyl-orthosilicate -TEOS, Si(OC2H5)4, (Sigma Aldrich), and sodium fluoride, NaF (Sigma Aldrich).

During the initial phase, the Fe(acac)_3_ solution was prepared by dissolution of 4 g of Fe(acac)_3_ in 50 mL of pure methanol. Obtained mixture was kept in contact and stirred for 30 min at 50 °C. The subsequent phase involved the dissolution of the silica precursor in 10 mL distilled water, alongside 10 mL of TEOS.

**Figure 8:**
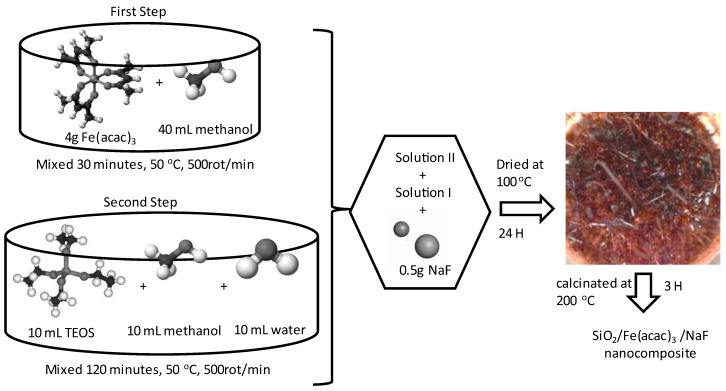
Schematic representation of SiO_2_/Fe(acac)_3_/NaF adsorbent material preparation.

Further, obtained solutions were mixed and kept in contact for 120 min at 50 °C by stirring the mixture at 400 rpm. After 1 h, 0.5 g of sodium fluoride, NaF (Sigma Aldrich), was added by mixing, which acted as a loosening agent for the gelled material. After one week of undergoing the aging process, the emergence of slender-needle-shaped crystals was observed. Resultant gel underwent a drying process for 24 h at a temperature of 100 °C, before being subjected to a subsequent heat treatment at 200 °C. Schematic representation of the adsorbent material preparation is presented in Figure 8.

In order to provide information regarding material surface morphology, but also to qualitatively confirm the composition of the synthesized material, scanning electron microscopy (SEM) and energy-dispersive X-ray spectroscopy (EDX) were performed by using the Quanta FEG 250 (FEI, Hillsbro, OR, USA) scanning electron microscope.

### 4.2. Selenium Adsorption Studies

#### 4.2.1. Kinetic and Thermodynamic Studies

In order to analyze Se(IV) adsorption kinetics, but also to understand the kinetic mechanism governing Se(IV) adsorption on the prepared adsorbent material, experimental data obtained were modeled using 3 different kinetic models: pseudo-first-order (Lagergren model) and pseudo-second-order kinetic model (Ho-McKay model), and intraparticle diffusion (Weber and Morris model).

To investigate the contact time and temperature impact adsorption capacity, a precisely measured quantity of 0.1 g of adsorbent material was utilized, onto which 25 mL of Se(IV) aqueous solution of an initial concentration of 100 µg L^−1^ was applied. Obtained samples were agitated at 200 rpm, for a range of time spanning 15, 30, 60, and 90 min, as well as at several temperatures (298 K, 308 K, 318 K, and 328 K) by using a thermostated bath.

#### 4.2.2. Kinetic Studies

The kinetic equations used to model the acquired experimental data for the pseudo-first-order equation (Lagergren model) [49] are as follows:

(1) $$\ln\left(q_{e}-q_{t}\right)=\ln q_{e}-k_{1}t$$

where q_e_—equilibrium adsorption capacity, µg g^−1^;
q_t_—adsorption capacity at t time, µg g^−1^;
k_1_—speed constant for pseudo-first order equation, min^−1^;
t—contact time, min.

The equation for pseudo-second-order model (model Ho and McKay) is as follows:

(2) $$\frac{1}{q_{t}}=\frac{1}{k_{2}q_{e}^{2}}+\frac{1}{q_{e}}$$

where q_e_—equilibrium adsoprtion capacity, µg g^−1^;
q_t_—adsorption capacity at t time, µg g^−1^;
k_2_—speed constant for pseudo-second-order equation, g µg^−1^ min^−1^;
t—contact time, min.

When the pseudo-first-order model was used, associated parameters (k_1_ and q_e,calc_) were obtained from the linear dependence between ln(q_e_ − q_t_) and time. Similarly, in the case of pseudo-second-order model usage, associated parameters (k_2_ and q_e,calc_) were evaluated based on linear dependence between t/q_t_ and t. In addition to these two typical kinetic models, obtained experimental data were also modeled by using an intraparticle diffusion model. In order to determine the controlling factor for adsorption speed, namely, to determine if it is governed by film or by intraparticle diffusion, recorded experimental data were subjected to analysis by using Weber and Morris model [50]:

(3) qt=kdifft12+C

where q_t_—adsorption capacity at t time, µg g^−1^;
k_diff_—speed constant for intraparticle diffusion, µg g^−1^ min^−0.5^;
C—constant correlated with the thickness of the liquid film surrounding the adsorbent particles.

#### 4.2.3. Thermodynamic Studies

All thermodynamic investigations were conducted over a temperature range of 298–328 K. The value of the Gibbs free energy was calculated by using the Gibbs–Helmholtz equation [51]:

(4) $$∆G^{0}=∆H^{0}+T∆S^{0}$$

where ΔG^0^—standard Gibbs free energy variation, J mol^−1^;
ΔH^0^—standard enthalpy variation, J mol^−1^;
ΔS^0^—standard entropy variation, J mol^−1^ K^−1^;
T—absolute temperature, K.

Values of entropy standard variation ΔS° and enthalpy standard variation ΔH° were computed by using the van’t Hoff equation and its linear from, which was obtained through representing ln K_d_ as function of 1/T.

(5) $$\ln k_{d}=\frac{∆S^{0}}{R}-\frac{∆H^{0}}{RT}$$

where K_d_—equilibrium constant;
ΔS^0^—standard entropy variation, J mol^−1^ K^−1^;
ΔH^0^—standard enthalpy variation, J mol^−1^;
R—the ideal gas constant, 8.314 J mol^−1^ K^−1^.

The proportional relationship between adsorption capacity at a specific state of equilibrium (q_e_) and the concentration at equilibrium (C_e_) represents the equilibrium constant.

(6) $$K_{d}=\frac{q_{e}}{C_{e}}$$

For the adsorption of Se(IV) on the SiO_2_/Fe(acac)_3_/NaF material, another aim was to ascertain the value of the activation energy E_a_ associated with studied adsorptive process. This value was evaluated by employing the Arrhenius equation in conjunction with the speed constant (k_2_) obtained through the application of pseudo-second-order kinetic model.

(7) $$\ln k_{2}=\ln A-\frac{E_{a}}{RT}$$

where k_2_—speed constant, g min^−1^ mg^−1^;
A—Arrhenius constant, g min mg^−1^;
E_a_—activation energy, kJ mol^−1^;
T—absolute temperature, K;
R—the ideal gas constant, 8.314 J mol^−1^ K^−1^.

The activation energy value was utilized to elucidate the character of the studied adsorption process, mainly to distinguish if Se(IV) adsorption occured through physical or chemical interactions.

#### 4.2.4. Equilibrium Studies

Another aim of the present study was to evaluate the impact of Se(IV)’s initial concentration on the adsorption capacity of the material SiO_2_/Fe(acac)_3_/NaF. In order to achieve this specific objective, solutions of varying Se(IV) concentrations were prepared (200, 400, 800, 1000, 2000, 3000, 5000, 7000, 8000, 10,000, 15,000, 20,000, 30,000, and 50,000 µg L^−1^). These concentrations were achieved by performing an appropriate dilution from a concentrated stock solution of 1000 mg L^−1^. Further, the adsorption tests were conducted at a pH value between 7 and 8, by keeping the studied Se(IV) solution in contact with adsorbent material at a temperature of 298 K for 60 min. Residual Se(IV) concentration was evaluated by using the atomic absorption spectrophotometer equipped with graphite furnace, AA 6800, Schimadzu (having a measurement error of 5%).

In order to determine the adsorption mechanism of Se(IV), obtained experimental data were further modeled by utilizing 3 different isotherms: Langmuir, Freundlich, and Sips. Langmuir isotherm [52] is used to evaluate the maximum adsorption capacity of used adsorbent material. The maximum adsorption capacity of prepared adsorbent material was acquired based on linear form of the Langmuir adsorption isotherm:

(8) $$\frac{C_{e}}{q_{e}}=\frac{1}{q_{L}K_{L}}+\frac{C_{e}}{q_{L}}$$

where q_L_—Langmuir maximum adsorption capacity, µg g^−1^;
K_L_—Langmuir constant.

The assumption of the Freundlich adsorption isotherm is that the adsorbent material surface is heterogeneous, so the heat distribution required for the studied adsorption process onto adsorbent material surface is uneven, leading to the occurrence of multilayer adsorption due to an unlimited number of active centers. Freundlich-isotherm-associated parameters were determined from linear form of Freundlich isotherm [53]:

(9) $${logq}_{e}=logK_{F}+1/n_{F}{logC}_{e}$$

where K_F_ and n_F_—characteristic constants that may be related to the relative adsorption capacity of the adsorbent and the adsorption intensity.

Sips adsorption isotherm [54] was inferred based on Langmuir and Freundlich isotherms. Within the case of low adsorbate concentrations, all the characteristics associated with Freundlich isotherm are present, and at higher adsorbate concentrations all the characteristics of Langmuir isotherm are present. Subsequently, this isotherm was used to calculate the adsorption capacity:

(10) $$q_{e}=\frac{q_{S}K_{S}C_{e}^{1/n_{S}}}{1+K_{s}C_{e}^{1/n_{S}}}$$

where K_S_—constant related to the adsorption capacity of the adsorbent;
n_S_—heterogeneity factor.

Temkin isotherm was developed in order to describe the adsorption on heterogeneous surfaces, surfaces characterized by a non-uniform distribution of sorption heat [55]. Another assumption of the Temkin model is that the heat of adsorption depends on temperature. Specific to this isotherm is the factor which takes into account the interaction established between the adsorbate and adsorbent, ignoring limit concentrations—low and large ones. Linear form of the Temkin model is

(11) $$q_{e}=\frac{RT}{b_{T}}\ln A_{T}+(\frac{RT}{b_{T}})\ln C_{e}$$

where A_T_—Temkin isotherm equilibrium binding constant (L/g), b_T_—Temkin isotherm constant, R—universal gas constant, and T—temperature. Temkin isotherm parameters were evaluated from the linear dependence between the quantity of sorbed ions (q_e_) and lnC_e_.

Dubinin–Radushkevich (DRK) isotherm represents a general and empirical model able to explain adsorption processes on heterogeneous surfaces with a Gaussian energy distribution, being related with the sorbent porous structure [56]. Linear form of the DRK isotherm is [57]

(12) $$\ln q_{e}=\ln q_{m}-\beta \varepsilon^{2}$$

where $\varepsilon =RT\ln\left(1+\frac{1}{C_{e}}\right)$, qm—maximum adsorption capacity (mg/g), β—activity coefficient constant related to sorption energy, and ε—Polanyi potential.

#### 4.2.5. pH Effect

pH’s impact on the Se(IV) adsorption on the prepared adsorbent material was studied by varying selenium solution pH in the range of 1–14. Hence, 0.1 g of adsorbent material was kept in contact at 298 K for 60 min with 25 mL of Se(IV) solution (SeO_2_ in HNO_3_ 0.5 mol L^−1^ Merck) of initial concentration C_0_ = 100 µg Se(IV) L^−1^. Selenium solution pH was adjusted to the desired range by using HNO_3_ and NaOH solutions, with concentrations in the range of 0.1–1 N. These solutions were prepared from HNO_3_ (63%, Carl Roth) and NaOH pellets (Sigma Aldrich).

## Figures and Tables

**Table 1:** Elemental composition of SiO_2_/Fe(acac)_3_/NaF material.

Elements	Wt,%	At,%
C	23.73	37.70
O	36.72	43.80
Fe	26.48	9.05
Na	2.31	1.89
F	1.43	1.21
Si	9.34	6.35
TOTAL	100	100

**Table 2:** Kinetic parameters for the adsorption of Se(IV) onto SiO_2_/Fe(acac)_3_/NaF.

Pseudo first order				
Temperature (K)	q_e,exp_ (µg/g)	k_1_ (min^−1^)	q_e,calc_ (µg/g)	R^2^
298	24.35	0.0383	10.07	0.9906
308	24.49	0.0386	14.78	0.9905
318	24.62	0.0388	17.64	0.9900
328	24.67	0.0371	20.61	0.9920
Pseudo second order				
Temperature (K)	q_e,exp_ (µg g^−1^)	k_2 _ (g µg^−1^∙min^−1^)	q_e,calc_ (µg g^−1^)	R^2^
298	24.35	1238.8	26.31	0.9997
308	24.49	1811.1	26.88	0.9996
318	24.62	2183.0	27.24	0.9997
328	24.67	3164.2	27.70	0.9996
Intraparticle diffusion model				
Temperature (K)	K_diff_ (mg·g^−1^ min^−1/2^)	C		R^2^
298	2.62	10.2		0.8792
308	2.88	10.9		0.8173
318	2.94	11.3		0.8019
328	2.98	12.01		0.8945

**Table 3:** Thermodynamic parameters for adsorption of Se(IV) onto SiO_2_/Fe(acac)_3_/NaF.

ΔH° (J mol^−1^)	ΔS° (J mol^−1^ K^−1^)	ΔG° (J mol^−1^)				R^2^
22.71	95.84	298 K	308 K	318 K	328 K	0.9899
		−28.53	−29.49	−30.45	−31.41	

**Table 4:** Parameters of isotherm model for adsorption of Se(IV) onto SiO_2_/Fe(acac)_3_/NaF.

Langmuir Isotherm			
q_m,exp_ (µg g^−1^)	K_L_ (L µg^−1^)	q_L_ (µg g^−1^)	R^2^
6180	6.2·10^−4^	7086	0.9810
Freundlich isotherm			
K_F_ (µg g^−1^)	1/n_F_		R^2^
208.2	0.35		0.8391
Sips isotherm			
K_S_	q_S_ (µg g^−1^)	1/n_S_	R^2^
1.62	6718	0.21	0.9916
Temkin isotherm			
A_T_ [L/g]	b_T_		R^2^
0.52	144.07		0.2788
Dubinin–Radushkevich isotherm			
q_m_ [mg/g]	ε		R^2^
1.01	0.366		0.5649

**Table 5:** Comparison of adsorption performance with other materials for Se(IV) adsorption.

Materials	q, mg g^−1^	References
Graphene oxide nanocomposite hydrogel beads	1.62	[36]
Fly ash extracted char carbon	0.68	[37]
Chitosan beads	2.00	[38]
Iron-Oxy hydroxides	0.001	[28]
Mesoporous activated alumina	0.0054	[39]
Fly ash extracted char carbon	0.44	[37]
Hematite	0.24	[40]
Magnetite	0.25	[41]
Iron-coated granular activated carbon	0.39	[42]
Corundum	0.59	[43]
Nano-Jacobsite	0.77	[44]
Aluminum-oxide-coated sand	0.92	[45]
Binary oxide [Fe (III)/SiO_2_]	1.33	[46]
Ferrihydrite	2.00	[47]
Chitosan beads	2.00	[38]
SiO_2_/Fe(acac)_3_/NaF	6.00	This paper

## References

[1] Zhang N. (2008). Selenite removal using GAC based iron-coated adsorbents. Civil and Environmental Engineering.

[2] Lakin H.W. (1973). Selenium in Our Environment. Trace Elements in the Environment.

[3] He Y., Xiang Y., Zhou Y., Yang Y., Zhang J., Huang H., Shang C., Luo L., Gao J., Tang L. (2018). Selenium contamination, consequences and remediation techniques in water and soils: A review. Environ. Res..

[4] Kalaitzidou K., Nikoletopoulos A.A., Bakouros L., Zouboulis A., Mitrakas M. (2020). Selenite Removal from Water. Environ. Sci. Proc..

[5] Pettine M., McDonald T.J., Sohn M., Anquandah G.A., Zboril R., Sharma V.K. (2015). A critical review of selenium analysis in natural water samples. Trends Environ. Anal. Chem..

[6] Tabelin C.B., Igarashi T., Villacorte-Tabelin M., Park I., Opiso E.M., Ito M., Hiroyoshi N. (2018). Arsenic, selenium, boron, lead, cadmium, copper, and zinc in naturally contaminated rocks: A review of their sources, modes of enrichment, mechanisms of release, and mitigation strategies. Sci. Total Environ..

[7] Etteieb S., Magdouli S., Zolfaghari M., Brar S. (2020). Monitoring and analysis of selenium as an emerging contaminant in mining industry: A critical review. Sci. Total Environ..

[8] Ali I., Shrivastava V. (2021). Recent advances in technologies for removal and recovery of selenium from (waste) water: A systematic review. J. Environ. Manag..

[9] He Y., Liu J., Han G., Chung T.-S. (2018). Novel thin-film composite nanofiltration membranes consisting of a zwitterionic co-polymer for selenium and arsenic removal. J. Membr. Sci..

[10] Yoon S., Cho K.-H., Kim M., Park S.-J., Lee C.-G., Choi N.-C. (2023). Selenium Removal from Aqueous Solution Using a Low-Cost Functional Ceramic Membrane Derived from Waste Cast Iron. Water.

[11] Opiso E.M., Sato T., Yoneda T. (2016). Immobilization of selenium by Mg-bearing minerals and its implications for selenium removal from contaminated water and wastewater. Appl. Clay Sci..

[12] Staicu L.C., Morin-Crini N., Crini G. (2017). Desulfurization: Critical step towards enhanced selenium removal from industrial effluents. Chemosphere.

[13] Kazeem T., Labaran B., Ahmed H.-U.-R., Mohammed T., Essa M., Al-Suwaiyan M., Vohra M. (2019). Treatment of Aqueous Selenocyanate Anions Using Electrocoagulation. Int. J. Electrochem. Sci..

[14] Baek K., Kasem N., Ciblak A., Vesper D., Padilla I., Alshawabkeh A.N. (2013). Electrochemical removal of selenate from aqueous solutions. Chem. Eng. J..

[15] Vohra M.S., Labaran B.A. (2020). Photocatalytic treatment of mixed selenocyanate and phenol streams: Process modeling, optimization, and kinetics. Environ. Prog. Sustain. Energy.

[16] Botlaguduru V.S.V., Batchelor B., Abdel-Wahab A. (2015). Application of UV–sulfite advanced reduction process to bromate removal. Remov. J. Water Process Eng..

[17] Vellanki B.P., Batchelor B. (2013). Perchlorate reduction by the sulfite/ultraviolet light advanced reduction process. J. Hazard. Mater..

[18] Liu X., Vellanki B.P., Batchelor B., Abdel-Wahab A. (2014). Degradation of 1,2-dichloroethane with advanced reduction processes (ARPs): Effects of process variables and mechanisms. Chem. Eng. J..

[19] Yoon S., Han D.S., Liu X., Batchelor B., Abdel-Wahab A. (2014). Degradation of 1,2-dichloroethane using advanced reduction processes. J. Environ. Chem. Eng..

[20] Zou R., Zhang H., Luo G., Fang C., Shi M., Hu H., Li X., Yao H. (2020). Selenium migration behaviors in wet flue gas desulfurization slurry and an in-situ treatment approach. Chem. Eng. J..

[21] Paul T., Saha N.C. (2019). Environmental Arsenic and Selenium Contamination and Approaches towards Its Bioremediation through the Exploration of Microbial Adaptations: A Review. Pedosphere.

[22] Hasanuzzaman M., Bhuyan M.H.M.B., Raza A., Hawrylak-Nowak B., Matraszek-Gawron R., Nahar K., Fujita M. (2020). Selenium Toxicity in Plants and Environment: Biogeochemistry and Remediation Possibilities. Plants.

[23] Fu Y., Wang J., Liu Q., Zeng H. (2014). Water-dispersible magnetic nanoparticle–graphene oxide composites for selenium removal. Carbon.

[24] Johansson C.L., Paul N.A., de Nys R., Roberts D.A. (2015). The complexity of biosorption treatments for oxyanions in a multi-element mine effluent. J. Environ. Manag..

[25] Gurunathan P., Hari S., Suseela S.B., Sankararajan R., Mukannan A. (2019). Production, characterization and effectiveness of cellulose acetate functionalized ZnO nanocomposite adsorbent for the removal of Se (VI) ions from aqueous media. Environ. Sci. Pollut. Res..

[26] Zhao Q., Huang J.-C., He S., Zhou W. (2020). Enhancement of a constructed wetland water treatment system for selenium removal. Sci. Total Environ..

[27] Zhao X., Zhang A., Zhang J., Wang Q., Huang X., Wu Y., Tang C. (2020). Enhanced Selenate Removal in Aqueous Phase by Copper-Coated Activated Carbon. Materials.

[28] Kalaitzidou K., Nikoletopoulos A.-A., Tsiftsakis N., Pinakidou F., Mitrakas M. (2019). Adsorption of Se(IV) and Se(VI) species by iron oxy-hydroxides: Effect of positive surface charge density. Sci. Total Environ..

[29] Vilardi G., Mpouras T., Dermatas D., Verdone N., Polydera A., Di Palma L. (2018). Nanomaterials application for heavy metals recovery from polluted water: The combination of nano zero-valent iron and carbon nanotubes. Competitive adsorption non-linear modeling. Chemosphere.

[30] Ji Y., Li L., Wang Y.-T. (2020). Selenium removal by activated alumina in batch and continuous-flow reactors. Water Environ. Res..

[31] Toyos-Rodríguez C., Calleja-García J., Torres-Sánchez L., López A., Abu-Dief A.M., Costa A., Elbaile L., Crespo R.D., Garitaonandia J.S., Lastra E. (2019). A Simple and Reliable Synthesis of Superparamagnetic Magnetite Nanoparticles by Thermal Decomposition of Fe(acac)_3_. J. Nanomater..

[32] Mladin G., Ciopec M., Negrea A., Duteanu N., Negrea P., Ianasi P., Ianasi C. (2022). Silica- Iron Oxide Nanocomposite Enhanced with Porogen Agent Used for Arsenic Removal. Materials.

[33] Shafiq I., Hussain M., Rashid R., Shafique S., Akhter P., Yang W., Ahmed A., Nawaz Z., Park Y.-K. (2021). Development of hierarchically porous LaVO4 for efficient visible-light-driven photocatalytic desulfurization of diesel. Chem. Eng. J..

[34] Zhang S., Ning S., Liu H., Zhou J., Wang S., Zhang W., Wang X., Wei Y. (2020). Highly-efficient separation and recovery of ruthenium from electroplating wastewater by a mesoporous silica-polymer based adsorbent. Microporous Mesoporous Mater..

[35] Zhang Y., Yu F., Cheng W., Wang J., Ma J. (2017). Adsorption Equilibrium and Kinetics of the Removal of Ammoniacal Nitrogen by Zeolite X/Activated Carbon Composite Synthesized from Elutrilithe. J. Chem..

[36] Bandara P.C., Perez J.V.D., Nadres E.T., Nannapaneni R.G., Krakowiak K.J., Rodrigues D.F. (2019). Graphene Oxide Nanocomposite Hydrogel Beads for Removal of Selenium in Contaminated Water. ACS Appl. Polym. Mater..

[37] Jegadeesan G.B., Mondal K., Lalvani S.B. (2015). Adsorption of Se(IV) and Se (VI) Using Copper-Impregnated Activated Carbon and Fly Ash-Extracted Char Carbon. Water Air Soil Pollut..

[38] Yamani J.S., Lounsbury A.W., Zimmerman J.B. (2014). Adsorption of selenite and selenate by nanocrystalline aluminum oxide, neat and impregnated in chitosan beads. Water Res..

[39] Meher A.K., Jadhav A., Labhsetwar N., Bansiwal A. (2019). Simultaneous removal of selenite and selenate from drinking water using mesoporous activated alumina. Appl. Water Sci..

[40] Rovira M., Giménez J., Martínez M., Martínez-Lladó X., de Pablo J., Martí V., Duro L. (2008). Sorption of selenium(IV) and selenium(VI) onto natural iron oxides: Goethite and hematite. J. Hazard. Mater..

[41] Martínez M., Giménez J., de Pablo J., Rovira M., and Duro L. (2006). Sorption of selenium(IV) and selenium(VI) onto magnetit. Appl. Surf. Sci..

[42] Zhang N., Gang D., Lin L.-S. (2010). Adsorptive Removal of Parts per Million Level Selenate Using Iron-Coated GAC Adsorbents. J. Environ. Eng..

[43] Peak D. (2006). Adsorption mechanisms of selenium oxyanions at the aluminum oxide/water interface. J. Colloid Interface Sci..

[44] Gonzalez C.M., Hernandez J., Parsons J.G., Gardea-Torresdey J.L. (2010). A study of the removal of selenite and selenate from aqueous solutions using a magnetic iron/manganese oxide nanomaterial and ICP-MS. Microchemical Journal.

[45] Kuan W.-H., Lo S.-L., Wang M.K., and Lin C.-F. (1998). Removal of Se(IV) and Se(VI) from water by aluminum-oxide-coated sand. Water Res..

[46] Chan Y.T., Kuan W.H., Chen T.Y., Wang M.K. (2009). Adsorption mechanism of selenate and selenite on the binary oxide systems. Water Res..

[47] Das S., Jim M.H., Essilfie-Dughan J. (2013). Adsorption of selenate onto ferrihydrite, goethite, and lepidocrocite under neutral pH conditions. Appl. Geochem..

[48] Khamkhash A., Srivastava V., Ghosh T., Akdogan G., Ganguli R., Aggarwal S. (2017). Mining-Related Selenium Contamination in Alaska, and the State of Current Knowledge. Minerals.

[49] Lagergren S. (1898). About the theory of so-called adsorption of soluble substabces. Sven. Vetenskapsakad. Handingarl.

[50] Weber W.J., Morris J.C. (1964). Equilibria and Capacities for Adsorption on Carbon. J. Sanit. Eng. Div..

[51] Atkins P., de Paula J. (2005). Atkins’ Physical Chemistry.

[52] Langmuir I. (1918). The adsorption of gases on plane surfaces of glass, mica and platinum. J. Am. Chem. Soc..

[53] Freundlich H.M.F. (1906). Over the adsorption in solution. J. Phys. Chem..

[54] Sips R. (1948). On the Structure of a Catalyst Surface. J. Chem. Phys..

[55] Erhayem M., Al-Tohami F., Mohamed R., Ahmida K. (2015). Isotherm, Kinetic and Thermodynamic Studies for the Sorption of Mercury (II) onto Activated Carbon from Rosmarinus officinalis Leaves. Am. J. Anal. Chem..

[56] Ayawei N., Ebelegi A.N., Wankasi D. (2017). Modelling and Interpretation of Adsorption Isotherms. J. Chem..

[57] Inyinbor A.A., Adekola F.A., Olatunji G.A. (2016). Kinetics, isotherms and thermodynamic modeling of liquid phase adsorption of Rhodamine B dye onto Raphia hookerie fruit epicarp. Water Resour. Ind..

